# Association between the atherogenic index of plasma and coronary collateral circulation in patients with chronic total occlusion

**DOI:** 10.1186/s12872-024-03992-y

**Published:** 2024-07-16

**Authors:** Shutong Dong, Jiaming Qiao, Ang Gao, Zehao Zhao, Xin Huang, Yi Kan, Zhiqiang Yang, Meishi Ma, Chu Fan, Hongya Han, Yujie Zhou

**Affiliations:** Department of Cardiology, Beijing Anzhen Hospital, Capital Medical University, Beijing Institute of Heart Lung and Blood Vessel Disease, Beijing Key Laboratory of Precision Medicine of Coronary Atherosclerotic Disease, Clinical Center for Coronary Heart Disease, Beijing, 100029 China

**Keywords:** Atherogenic index of plasma, Coronary artery disease, Chronic total occlusion, Coronary collateral circulation, Triglyceride, High-density lipoprotein cholesterol

## Abstract

**Background:**

The atherogenic index of plasma (AIP) is considered an independent risk factor for coronary artery disease (CAD). The present study investigated whether AIP correlates with the formation of coronary collateral circulation (CCC) in CAD patients with chronic total occlusion (CTO).

**Methods:**

This retrospective study included 1093 CAD patients with CTO confirmed by coronary angiography from January 2020 to December 2020 at Beijing Anzhen Hospital. Based on the Rentrop scoring system, the patients were divided into the good CCC group and the poor CCC group. AIP was calculated by log (triglyceride/high-density lipoprotein cholesterol). Meanwhile, the study population was further divided into four groups according to the quartiles of AIP.

**Results:**

Patients in the poor CCC group exhibited significantly higher AIP compared to those in the good CCC group (0.31 ± 0.27 vs. 0.14 ± 0.24, *p* < 0.001). Multivariate logistic regression analysis revealed an independent association between AIP and poor CCC, regardless of whether AIP was treated as a continuous or categorical variable (*p* < 0.001), after adjusting for confounding factors. Besides, this association remained consistent across most subgroups. The incorporation of AIP into the baseline model significantly enhanced the accuracy of identifying poor CCC [area under the curve (AUC): baseline model, 0.661 vs. baseline model + AIP, 0.721, *p* for comparison < 0.001].

**Conclusions:**

Elevated AIP is independently associated with an increased risk of poor CCC in CAD patients with CTO, and AIP may improve the ability to identify poor CCC in clinical practice.

## Introduction

Coronary artery disease (CAD) is a major contributor to the global burden of disease and remains one of the leading causes of mortality. Statistics indicate that around 9.44 million people globally succumbed to CAD in 2021, presenting a considerable public health and socioeconomic challenge [1]. Advancements in treatment technology have significantly reduced mortality and improved long-term outcomes in patients with CAD through revascularization such as percutaneous coronary intervention (PCI) and coronary artery bypass grafting (CABG). Nonetheless, many patients experience incomplete revascularization or fall into the category of “no-option” individuals, characterized by being ineligible for PCI or CABG with persistent angina symptoms despite optimal medical therapy, resulting in a significantly compromised quality of life [2]. Challenges in achieving complete revascularization among patients with refractory angina primarily stem from advanced age, comorbidities, chronic total occlusion (CTO), multivessel disease, diffuse lesions, and low ejection fraction [3].

CTO represents the ultimate obstacle to current interventional therapies. Previous studies have shown that CTO-PCI carries a lower success rate and higher complication rate compared to non-CTO PCI. Additionally, CTO-PCI has not demonstrated a significant benefit in reducing major adverse cardiovascular events (MACE) compared with optimal medical therapy [4, 5]. The guidelines for coronary artery revascularization have downgraded the recommendation for CTO-PCI from class IIa to class IIb due to insufficient strong clinical evidence [6]. Hence, there is a necessity to investigate other therapeutic modalities for this population.

Coronary collateral circulation (CCC) serves as an anastomotic branch connecting the epicardial coronary arteries, offering an alternative blood supply to the ischemic myocardium through collateral vessel remodeling and dilatation in the presence of epicardial coronary artery stenosis [7]. Extensive evidence indicates that well-developed CCC exerts a protective effect, leading to reduced myocardial infarct size, decreased formation of ventricular aneurysms, and improved survival rates compared to poor CCC [8, 9]. Hence, the formation of well-developed CCC may emerge as a promising therapeutic strategy for patients with CTO.

Various factors can affect the formation of CCC, yet there exists no reliable clinical indicator to assess its degree of development. Previous studies have demonstrated a close association between poor CCC and disorders of glucose and lipid metabolism [10, 11]. The atherogenic index of plasma (AIP), calculated based on triglyceride (TG) and high-density lipoprotein cholesterol (HDL-C), is an effective surrogate marker for the assessment of lipid-related risk and atherosclerosis [12, 13]. Besides, AIP serves as an independent risk factor for hypertension, type 2 diabetes mellitus (T2DM), metabolic syndrome (MetS), CAD, and cardiovascular (CV) events [14–16], and has also been found to be closely related to the progression of CTO [17, 18]. To date, no study has explored the correlation between AIP and CCC in CAD patients with CTO. Therefore, the present study aims to fill this knowledge gap and examine its potential mechanism, thereby providing a convenient indicator for the clinical assessment of CCC, which holds great practical implications.

## Methods

### Study population

**Fig. 1:**
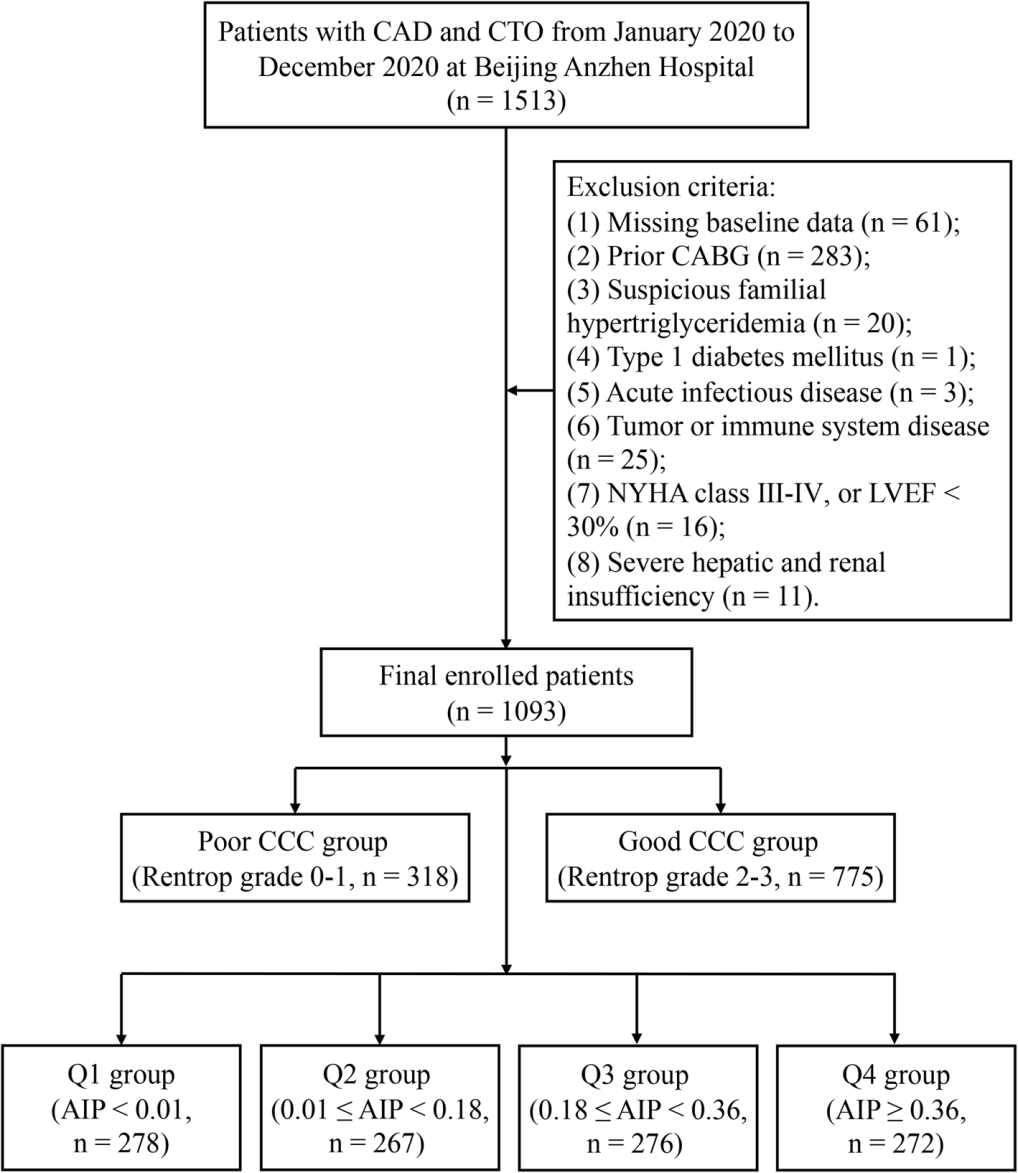
Flow chart for the enrollment of the study population. CAD, coronary artery disease; CTO, chronic total occlusion; CABG, coronary artery bypass grafting; NYHA, New York Heart Association; LVEF, left ventricular ejection fraction; CCC, coronary collateral circulation; AIP, atherogenic index of plasma

This is a single-center, observational, and retrospective study. A total of 1513 patients diagnosed with CAD and presenting CTO in more than one major branch of the coronary artery were included in this study, with confirmation by coronary angiography at Beijing Anzhen Hospital from January 2020 to December 2020. CTO refers to 100% stenosis with thrombolysis in myocardial infarction (TIMI) grade 0 flow for more than three months [19]. The exclusion criteria were as follows: (1) missing baseline data; (2) previous history of CABG; (3) suspicious familial hypertriglyceridemia; (4) type 1 diabetes mellitus; (5) acute infectious diseases; (6) tumor or immune system disease; (7) New York Heart Association (NYHA) class III-IV, or left ventricular ejection fraction (LVEF) < 30%; and (8) severe hepatic and renal insufficiency. Finally, 1093 CAD patients with CTO were enrolled in the present analysis, as depicted in Fig. 1. The study was approved by the Ethics Committee of Beijing Anzhen Hospital and strictly followed the Declaration of Helsinki. The Ethics Committee of Beijing Anzhen Hospital, Capital Medical University waived the requirement for informed consent due to the retrospective nature of the study. All patients’ identity information has been removed.

### Data collection and definitions

Basic clinical information, including age, sex, body mass index (BMI), smoking history, medical history, laboratory measurements, and angiographic results, was collected from the medical record management system of Beijing Anzhen Hospital. LVEF was determined using Doppler echocardiography by experienced echocardiographers at admission. The following laboratory measurements were determined by standard techniques in the central laboratory of Beijing Anzhen Hospital, using blood samples extracted after fasting for more than 8 h: high-sensitivity C-reactive protein (hs-CRP), creatinine, urea, uric acid, fasting blood glucose (FBG), glycosylated albumin (GA), glycosylated hemoglobin A1c (HbA1c), TG, total cholesterol (TC), low-density lipoprotein cholesterol (LDL-C), and HDL-C. AIP was calculated as follows: AIP = log (TG [mmol/L]/HDL-C [mmol/L]) [12]. Non-HDL-C was calculated as follows: non-HDL-C = TC (mmol/L) – HDL-C (mmol/L). Castelli’s risk index (CRI) was calculated as follows: CRI-I = TC (mmol/L)/HDL-C (mmol/L). CRI-II = LDL-C (mmol/L)/HDL-C (mmol/L) [20].

Hypertension was defined as a previous diagnosis of hypertension or use of antihypertensive medication, or a new diagnosis of hypertension after admission based on the following criteria: repeated office systolic blood pressure (SBP) ≥ 140 mmHg and/or diastolic blood pressure (DBP) ≥ 90 mmHg [21]. T2DM was defined as a previous diagnosis of T2DM or use of glucose-lowering medication, or a new diagnosis of T2DM after admission based on the following criteria: (1) FBG ≥ 7.0 mmol/L (126 mg/dL); (2) 2-h blood glucose ≥ 11.1 mmol/L (200 mg/dL) during 75 g oral glucose tolerance test (OGTT); (3) HbA1c ≥ 6.5% (48 mmol/mol); and (4) classic symptoms of hyperglycemia or hyperglycemic crisis, a random blood glucose ≥ 11.1 mmol/L (200 mg/dL) [22]. Dyslipidemia was defined as a previous diagnosis of dyslipidemia or use of lipid-lowering medication, or a new diagnosis of dyslipidemia after admission based on the following criteria: (1) TC ≥ 5.2 mmol/L, (2) HDL-C < 1.0 mmol/L, (3) LDL-C ≥ 3.4 mmol/L, and (4) TG ≥ 1.7 mmol/L [23].

### Procedure and evaluation of coronary collateral circulation

CCC in CTO was assessed by coronary angiography performed in the selected population via the radial or femoral artery with 6F or 7F angiography catheters. The procedure results were interpreted and recorded by at least two professional and independent interventional cardiologists who were unaware of the purpose of this study. CCC was graded according to the Rentrop scoring system as follows: Rentrop grade 0, no visible collateral circulation filling; Rentrop grade 1, collateral circulation filling of branches of the vessel to be dilated but the epicardial segment was not visible; Rentrop grade 2, partial collateral circulation filling of the epicardial segment of the vessel to be dilated; Rentrop grade 3, complete collateral circulation filling of the epicardial segment of the vessel to be dilated [24]. The collateral vessel with the highest Rentrop grade was used for the final analysis when patients had more than one collateral vessel. Moreover, patients were further divided into the good CCC group (Rentrop grade 2–3) and the poor CCC group (Rentrop grade 0–1) based on the Rentrop scoring system.

### Statistical analysis

Continuous variables with a normal distribution were presented as the mean ± standard deviation (SD), and the independent sample t-test was used for comparisons between the groups. Medians with interquartile ranges (IQR) were used for continuous variables that did not conform to a normal distribution, and the Mann–Whitney U test was used for comparisons between the groups. Categorical variables were expressed as frequencies (percentages), and comparisons between the groups were performed by Pearson’s chi-squared test. The correlation between the AIP and cardiovascular risk factors was evaluated using either the Pearson correlation test or Spearman’s rank sum test. Univariate logistic regression analysis was applied to identify risk factors that affected CCC in CAD patients with CTO. Variables with significant differences in the univariate analysis or clinical significance were included in the multivariate logistic regression analysis to further assess whether AIP was an independent risk factor for poor CCC. The results were displayed as the odds ratio (OR) with 95% confidence interval (CI).

To control for the influence of confounding factors on the results of this study, three models were constructed to evaluate the correlation between AIP and CCC. Model 1 was adjusted for age, sex, BMI, current smoking, hypertension, T2DM, and dyslipidemia; Model 2 was adjusted for variables of model 1 and hs-CRP, creatinine, uric acid, FBG, HbA1c, TC, LDL-C, and LVEF; Model 3 was adjusted for variables of model 2 and the number of lesions and vessels with CTO. Besides, AIP was presented as a continuous or categorical variable to validate the independent association between AIP and poor CCC in each model. The restricted cubic spline (RCS) curve was constructed to illustrate the linear or non-linear relationship between AIP and poor CCC with the adjustment of Model 3. Receiver operating characteristic (ROC) curves with DeLong’s test were used to calculate the area under the curve (AUC) and the optimal cut-off value to further evaluate the incremental effect of AIP on discrimination capacity beyond the baseline model, including age, sex, BMI, current smoking, hypertension, T2DM, dyslipidemia, hs-CRP, creatinine, uric acid, FBG, HbA1c, LVEF, number of lesions, and vessel with CTO. Meanwhile, the net reclassification improvement (NRI) and integrated discrimination improvement (IDI) were tested to explore the discrimination capacity of AIP for identifying poor CCC. Furthermore, subgroup analysis was performed to investigate whether the evaluation value of the AIP for poor CCC was consistent across subgroups.

All statistical analyses were conducted using SPSS 26.0 (IBM Corporation, IL, USA) and R Programming Language 4.2.1 (Vienna, Austria). All *p*-values were two-tailed, and a *p*-value < 0.05 was considered statistically significant.

## Results

### Baseline characteristics

The final analysis enrolled 1093 participants with a mean age of 58.8 ± 10.2 years, of which 936 (85.6%) were male. Based on the Rentrop scoring system, patients were divided into the good CCC group (Rentrop grade 2–3, *n* = 775) and the poor CCC group (Rentrop grade 0–1, *n* = 318). The baseline characteristics of the demographics, medical history, laboratory measurements, and angiographic results are shown in Table 1. Compared with the good CCC group, the poor CCC group had a higher level of AIP (0.31 ± 0.27 vs. 0.14 ± 0.24, *p* < 0.001). In addition, the poor CCC group had a higher proportion of individuals with high BMI and dyslipidemia than the good CCC group. Despite the lack of significant differences, the poor CCC group had more current smokers and more patients with T2DM and three-vessel disease compared with the good CCC group. In terms of laboratory measurements, patients in the poor CCC group had higher levels of hs-CRP, uric acid, FBG, HbA1c, TG, TC, LDL-C, and non-HDL-C, but lower levels of HDL-C compared to those in the good CCC group. Besides, the left anterior descending artery (LAD) with CTO tended to form poor CCC. There were no significant differences in other characteristics between the two groups.

**Table 1:** Baseline characteristics of the total population stratified by CCC group

Characteristics	Total population (*n* = 1093)	Good CCC group (Rentrop grade 2–3) (*n* = 775)	Poor CCC group (Rentrop grade 0–1) (*n* = 318)	*p* value
**Demographics**				
Age, years	58.8 ± 10.2	59.1 ± 10.2	58.0 ± 10.1	0.100
Male	936 (85.6%)	666 (85.9%)	270 (84.9%)	0.729
BMI, kg/m^2^	26.36 ± 3.30	26.23 ± 3.32	26.70 ± 3.21	0.032
Current Smoking	597 (54.6%)	409 (52.8%)	188 (59.1%)	0.065
**Medical history**				
Hypertension	734 (67.2%)	528 (68.1%)	206 (64.8%)	0.317
T2DM	463 (42.4%)	316 (40.8%)	147 (46.2%)	0.112
Dyslipidemia	900 (82.3%)	620 (80.0%)	280 (88.1%)	0.002
**Laboratory measurements**				
hs-CRP, mg/L	1.02 (0.52, 2.63)	0.97 (0.48, 2.37)	1.12 (0.68, 3.14)	0.002
Creatinine, mmol/L	78.34 ± 19.61	77.98 ± 18.89	79.22 ± 21.28	0.342
Urea, mmol/L	5.59 ± 1.83	5.52 ± 1.80	5.75 ± 1.90	0.057
Uric acid, mmol/L	355.54 ± 94.99	349.57 ± 93.32	370.11 ± 97.56	0.001
FBG, mmol/L	5.88 (5.11, 7.24)	5.75 (5.06, 7.00)	6.22 (5.22, 8.10)	< 0.001
GA, %	14.39 (12.96, 17.16)	14.37 (13.00, 16.80)	14.47 (12.91, 17.93)	0.455
HbA1c, %	6.10 (5.70, 6.90)	6.10 (5.70, 6.75)	6.20 (5.80, 7.38)	0.001
TG, mmol/L	1.5 (1.1, 2.1)	1.4 (1.1, 1.8)	2.1 (1.3, 2.8)	< 0.001
TC, mmol/L	3.92 ± 0.99	3.83 ± 0.94	4.14 ± 1.07	< 0.001
LDL-C, mmol/L	2.27 ± 0.88	2.22 ± 0.85	2.39 ± 0.95	0.004
HDL-C, mmol/L	1.04 ± 0.25	1.06 ± 0.25	0.99 ± 0.24	< 0.001
Non-HDL-C, mmol/L	2.88 ± 0.97	2.77 ± 0.91	3.14 ± 1.06	< 0.001
AIP	0.19 ± 0.26	0.14 ± 0.24	0.31 ± 0.27	< 0.001
LVEF, %	59 ± 8	59 ± 8	59 ± 8	0.389
**Angiographic results**				
Number of lesions				0.912
One-vessel disease	154 (14.1%)	111 (14.3%)	43 (13.5%)	0.803
Two-vessel disease	313 (28.6%)	223 (28.8%)	90 (28.3%)	0.934
Three-vessel disease	626 (57.3%)	441 (56.9%)	185 (58.2%)	0.750
Vessels with CTO				0.078
LAD	373 (34.1%)	249 (32.1%)	124 (39.0%)	0.035
LCX	192 (17.6%)	137 (17.7%)	55 (17.3%)	0.950
RCA	528 (48.3%)	389 (50.2%)	139 (43.7%)	0.060

*CCC* Coronary collateral circulation, *BMI* Body mass index, *T2DM* Type 2 diabetes mellitus, *hs-CRP* High-sensitivity C-reactive protein, *FBG* Fasting blood glucose, *GA* Glycated albumin, *HbA1c* Glycosylated hemoglobin A1c, *TG* Triglyceride, *TC* Total cholesterol, *LDL-C* Low-density lipoprotein cholesterol, *HDL-C* High-density lipoprotein cholesterol, *AIP* Atherogenic index of plasma, *LVEF* Left ventricular ejection fraction, *CTO* Chronic total occlusion, *LAD* Left anterior descending artery, *LCX* Left circumflex artery, *RCA* Right coronary artery

Meanwhile, patients were divided into four groups according to the quartiles of AIP: Q1 group (AIP < 0.01, *n* = 278), Q2 group (0.01 ≤ AIP < 0.18, *n* = 267), Q3 group (0.18 ≤ AIP < 0.36, *n* = 276), and Q4 group (AIP ≥ 0.36, *n* = 272). The baseline characteristics of the total population stratified by the quartiles of AIP are shown in Table 2. Patients with higher levels of AIP tended to be younger, have higher BMI, and exhibit a greater prevalence of dyslipidemia. Moreover, the levels of hs-CRP, creatinine, urea, uric acid, FBG, HbA1c, TG, TC, LDL-C, and non-HDL-C in the higher AIP group were significantly increased, while the level of HDL-C was significantly decreased compared with those in the lower AIP group. Other characteristics between the two groups were not significantly different.

**Table 2:** Baseline characteristics of the total population stratified by the quartiles of AIP

Characteristics	Total population (*n* = 1093)	Q1 group (*n* = 278)	Q2 group (*n* = 267)	Q3 group (*n* = 276)	Q4 group (*n* = 272)	*p* value
**Demographics**						
Age, years	58.8 ± 10.2	61.3 ± 9.3	59.4 ± 9.7	57.8 ± 10.3	56.5 ± 10.8	< 0.001
Male	936 (85.6%)	228 (82.0%)	231 (86.5%)	234 (84.8%)	243 (89.3%)	0.097
BMI, kg/m^2^	26.36 ± 3.30	25.29 ± 3.47	26.12 ± 2.74	26.78 ± 3.44	27.29 ± 3.14	< 0.001
Current Smoking	597 (54.6%)	139 (50.0%)	139 (52.1%)	154 (55.8%)	165 (60.7%)	0.064
**Medical history**						
Hypertension	734 (67.2%)	176 (63.3%)	188 (70.4%)	182 (65.9%)	188 (69.1%)	0.283
T2DM	463 (42.4%)	100 (36.0%)	115 (43.1%)	126 (45.7%)	122 (44.9%)	0.085
Dyslipidemia	900 (82.3%)	167 (60.1%)	194 (72.7%)	268 (97.1%)	271 (99.6%)	< 0.001
**Laboratory measurements**						
hs-CRP, mg/L	1.02 (0.52, 2.63)	0.77 (0.42, 1.86)	0.89 (0.51, 2.08)	1.12 (0.60, 3.02)	1.29 (0.75, 3.31)	< 0.001
Creatinine, mmol/L	78.34 ± 19.61	75.20 ± 16.76	77.41 ± 18.50	78.64 ± 19.18 ±	82.16 ± 22.97	< 0.001
Urea, mmol/L	5.59 ± 1.83	5.56 ± 1.71	5.45 ± 1.69	5.41 ± 1.49	5.94 ± 2.29	0.003
Uric acid, mmol/L	355.54 ± 94.99	320.24 ± 81.43	348.32 ± 88.61	355.67 ± 91.17	398.58 ± 101.24	< 0.001
FBG, mmol/L	5.88 (5.11, 7.24)	5.57 (5.00, 6.58)	5.75 (5.11, 6.94)	6.30 (5.12, 7.88)	6.06 (5.21, 7.70)	< 0.001
GA, %	14.39 (12.96, 17.16)	14.50 (13.23, 16.51)	14.40 (12.96, 16.99)	14.28 (12.90, 17.78)	14.30 (12.75, 17.44)	0.834
HbA1c, %	6.10 (5.70, 6.90)	5.90 (5.60, 6.60)	6.10 (5.70, 7.00)	6.20 (5.70, 6.90)	6.20 (5.80, 7.20)	< 0.001
TG, mmol/L	1.76 ± 0.91	0.94 ± 0.23	1.36 ± 0.26	1.79 ± 0.35	2.96 ± 0.89	< 0.001
TC, mmol/L	3.92 ± 0.99	3.73 ± 0.92	3.82 ± 0.96	3.92 ± 0.96	4.20 ± 1.07	< 0.001
LDL-C, mmol/L	2.27 ± 0.88	2.11 ± 0.83	2.25 ± 0.88	2.32 ± 0.85	2.38 ± 0.95	0.002
HDL-C, mmol/L	1.04 ± 0.25	1.26 ± 0.25	1.08 ± 0.18	0.97 ± 0.18	0.85 ± 0.15	< 0.001
Non-HDL-C, mmol/L	2.88 ± 0.97	2.47 ± 0.86	2.74 ± 0.88	2.95 ± 0.89	3.36 ± 1.02	< 0.001
AIP	0.19 ± 0.26	-0.13 ± 0.12	0.10 ± 0.05	0.27 ± 0.05	0.53 ± 0.13	< 0.001
LVEF, %	59 ± 8	60 ± 7	59 ± 9	59 ± 8	59 ± 8	0.584
**Angiographic results**						
Number of lesions						0.376
One-vessel disease	154 (14.1%)	39 (14.0%)	45 (16.9%)	36 (13.0%)	34 (12.5%)	0.475
Two-vessel disease	313 (28.6%)	83 (29.9%)	82 (30.7%)	80 (29.0%)	68 (25.0%)	0.468
Three-vessel disease	626 (57.3%)	156 (56.1%)	140 (52.4%)	160 (58.0%)	170 (62.5%)	0.122
Vessels with CTO						0.291
LAD	373 (34.1%)	99 (35.6%)	99 (37.1%)	88 (31.9%)	87 (32.0%)	0.479
LCX	192 (17.6%)	43 (15.5%)	54 (20.2%)	53 (19.2%)	42 (15.4%)	0.320
RCA	528 (48.3%)	136 (48.9%)	114 (42.7%)	135 (48.9%)	143 (52.6%)	0.143

All abbreviations are as in Table 1

### Correlation between AIP and cardiovascular risk factors

Correlation analysis demonstrated that AIP was related to various cardiovascular risk factors. As shown in Table 3, AIP had positive associations with males, BMI, current smoking, T2DM, dyslipidemia, hs-CRP, creatinine, uric acid, FBG, HbA1c, TG, TC, LDL-C, and non-HDL-C, while negative associations with age and HDL-C (all *p* < 0.05).

**Table 3:** Correlation between AIP and cardiovascular risk factors

Variable	Correlation coefficient	*p* value
Age	-0.177	< 0.001
Male	0.065	0.033
BMI	0.233	< 0.001
Current smoking	0.088	0.004
Hypertension	0.037	0.218
T2DM	0.076	0.012
Dyslipidemia	0.441	< 0.001
hs-CRP	0.197	< 0.001
Creatinine	0.143	< 0.001
Uric acid	0.312	< 0.001
FBG	0.149	< 0.001
HbA1c	0.156	< 0.001
TG	0.891	< 0.001
TC	0.178	< 0.001
LDL-C	0.107	< 0.001
HDL-C	-0.663	< 0.001
Non-HDL-C	0.352	< 0.001
LVEF	0.006	0.836

All abbreviations are as in Table 1

### Association between AIP and coronary collateral circulation

**Fig. 2:**
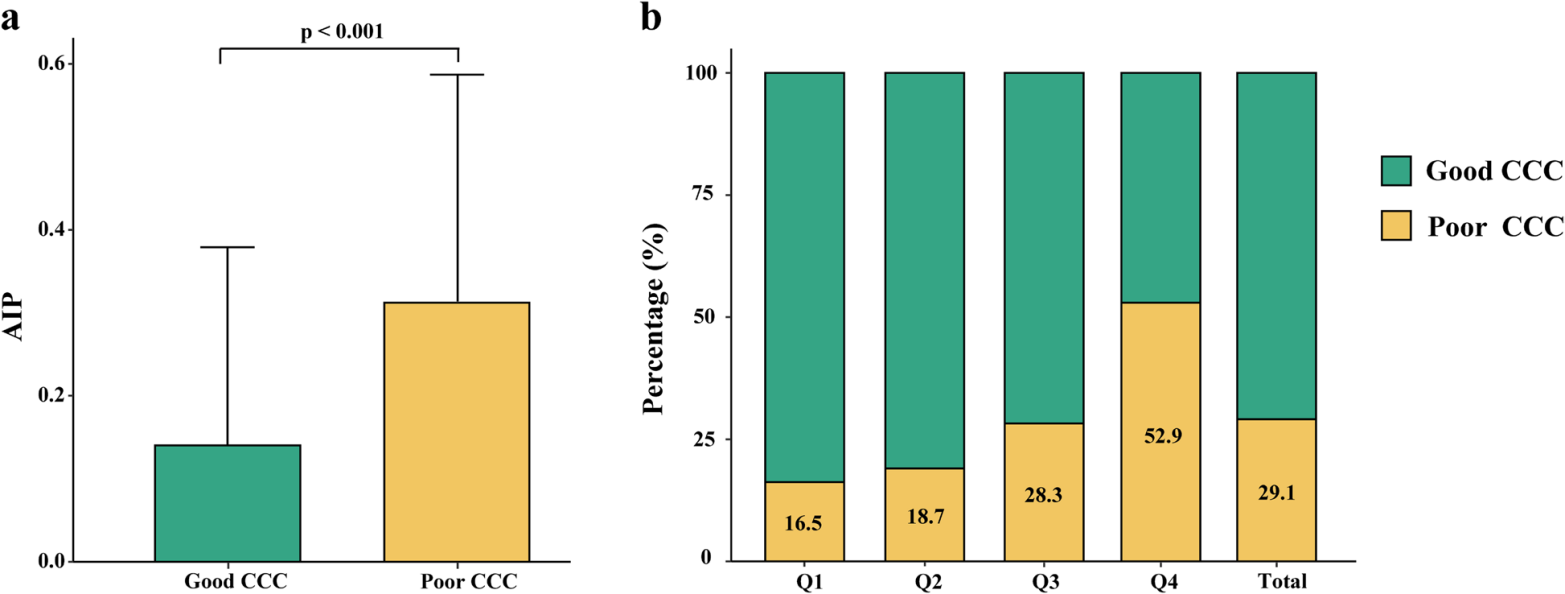
Association between AIP and CCC. **a** comparison of AIP between the good CCC group and poor CCC group; **b** prevalence of poor CCC according to the quartiles of AIP AIP, atherogenic index of plasma; CCC, coronary collateral circulation

Figure 2a was performed to compare the difference in AIP between the poor CCC group and the good CCC group. It showed that AIP was significantly higher in the poor CCC group than those in the good CCC group (*p* < 0.001). Furthermore, we found that the prevalence of poor CCC was significantly higher in the higher AIP group. The percentages of poor CCC according to the quartiles of AIP were 16.5%, 18.7%, 28.3%, and 52.9%, respectively (*p* < 0.001), which increased stepwise from the lowest AIP quartile to the highest quartile (Fig. 2b).

Multivariate logistic regression analysis showed that AIP was an independent risk factor for poor CCC, regardless of whether AIP was a continuous or categorical variable (Table 4). When AIP was considered a continuous variable, each 1-unit higher AIP was independently associated with an elevated risk of poor CCC after adjustment with different models (all *p* < 0.001). When AIP was considered a categorical variable, an increasing risk for poor CCC was observed with increasing AIP after adjusting for different models (all *p* for trend < 0.001). Moreover, the risk of poor CCC was significantly increased in the Q3 group and Q4 group compared with the Q1 group (all *p* < 0.05), but not in the Q2 group (Table 4).

**Table 4:** Predictive value of AIP for poor CCC in different models

	**OR**	**95% CI**	***p* value**	***p* for trend**
**Crude model**				
AIP as a categorical variable^a^				< 0.001
Q1	ref	ref	ref	
Q2	1.16	0.75–1.81	0.504	
Q3	1.99	1.32–3.01	0.001	
Q4	5.67	3.85–8.50	< 0.001	
AIP as a continuous variable^b^	15.07	8.66–26.69	< 0.001	
**Model 1**				
AIP as a categorical variable^a^				< 0.001
Q1	ref	ref	ref	
Q2	1.20	0.77–1.89	0.421	
Q3	2.07	1.33–3.29	0.002	
Q4	6.09	3.92–9.64	< 0.001	
AIP as a continuous variable^b^	17.61	9.38–33.80	< 0.001	
**Model 2**				
AIP as a categorical variable^a^				< 0.001
Q1	ref	ref	ref	
Q2	1.11	0.70–1.76	0.662	
Q3	1.71	1.07–2.75	0.025	
Q4	4.41	2.75–7.15	< 0.001	
AIP as a continuous variable^b^	10.11	5.09–20.51	< 0.001	
**Model 3**				
AIP as a categorical variable^a^				< 0.001
Q1	ref	ref	ref	
Q2	1.08	0.68–1.72	0.742	
Q3	1.71	1.07–2.75	0.026	
Q4	4.47	2.79–7.28	< 0.001	
AIP as a continuous variable^b^	10.42	5.21–21.31	< 0.001	

Model 1: adjusted for age, male, BMI, current smoking, hypertension, T2DM, and dyslipidemia

Model 2: adjusted for variables in Model 1 and hs-CRP, creatinine, uric acid, FBG, HbA1c, TC, LDL-C, and LVEF

Model 3: adjusted for variables in Model 2 and number of lesions and vessels with CTO

^a^The HR was examined regarding the Q1 group as a reference

^b^The HR was examined by per 1-unit increase of AIP

*OR* Odds ratio, *CI* Confidence interval, *ref* reference, other abbreviations are as in Table 1

**Fig. 3:**
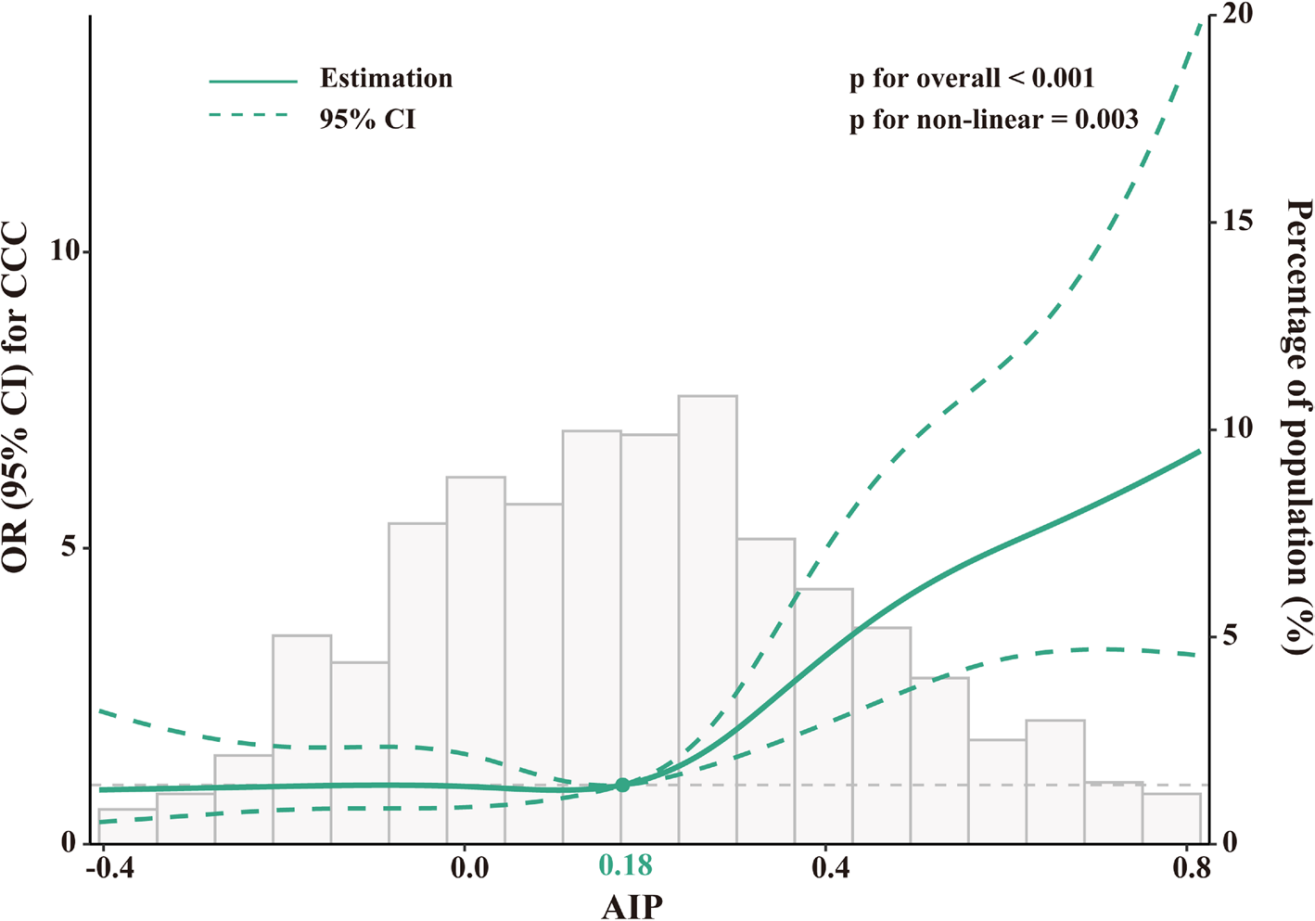
RCS curve for the association of AIP with CCC. The green solid line represents the OR, and the green dashed line represents the 95% CI. The RCS analysis was performed by using Model 3 (adjusted for age, male, BMI, current smoking, hypertension, T2DM, dyslipidemia, hs-CRP, creatinine, uric acid, FBG, HbA1c, TC, LDL-C, LVEF, number of lesions and vessels with CTO). The OR was examined by per 1-unit increase of AIP. AIP, atherogenic index of plasma; CCC, coronary collateral circulation; OR, odds ratio; CI, confidence interval; other abbreviations are as in Table 1.

Subsequently, the RCS curve revealed a non-linear relationship between AIP and poor CCC with the adjustment of Model 3. The risk of poor CCC was relatively flat until approximately 0.18 of AIP, beyond which it exhibited a rapid increase (*p* for non-linear = 0.003, *p* for overall < 0.001) (Fig. 3).

### Incremental effect of AIP on coronary collateral circulation

**Fig. 4:**
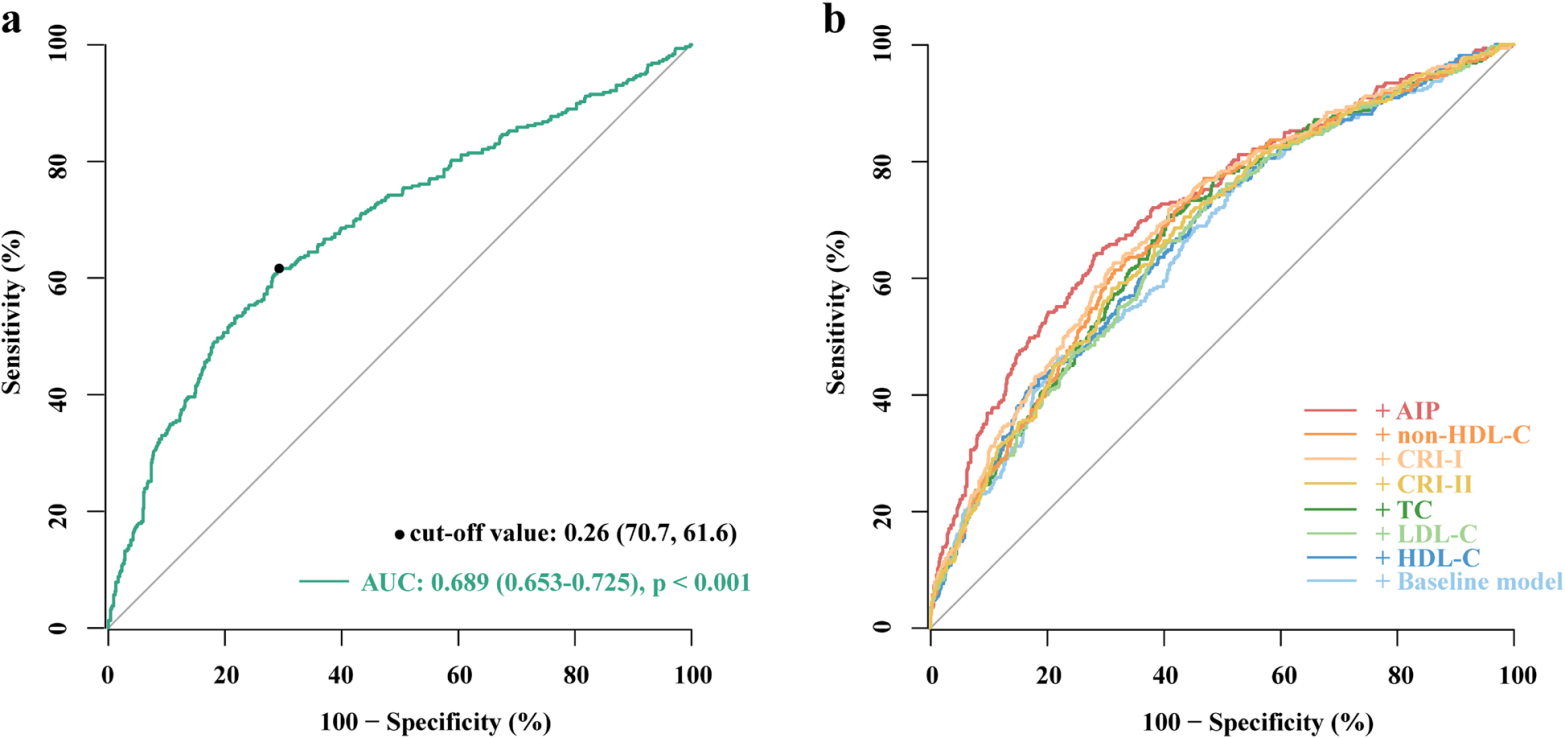
ROC curves evaluating diagnostic performance of AIP and other indicators for poor CCC. **a** the ROC curve of AIP for poor CCC; **b** the discriminative value of different models for evaluating CCC using ROC curve. The baseline model included age, male, BMI, current smoking, hypertension, T2DM, dyslipidemia, hs-CRP, creatinine, uric acid, FBG, HbA1c, LVEF, number of lesions, and vessels with CTO. AUC, area under the curve; AIP, atherogenic index of plasma; CRI, Castelli’s risk index; other abbreviations are as in Table 1

The ROC curve showed that the optimal cut-off value of AIP for identifying poor CCC was 0.26, with a sensitivity of 61.6% and a specificity of 70.7%. The AUC of AIP for identifying poor CCC was 0.689 (95% CI 0.653–0.725, *p* < 0.001) (Fig. 4a). As shown in Fig. 4b and Table 5, incorporating AIP into the baseline model had the most significant incremental effect for assessing the status of CCC compared with other indicators (AUC: baseline model, 0.661 vs. baseline model + AIP, 0.721, *p* for comparison < 0.001). In addition, IDI and NRI demonstrated that the inclusion of AIP compared with other indicators had the maximum enhancement to identify poor CCC based on the baseline model, with an NRI of 0.455 and an IDI of 0.063 (all *p* < 0.001) (Table 5).

**Table 5:** Incremental effect of AIP and other indicators for identifying poor CCC

	**AUC**	**95% CI**	***p* value**	**NRI**	**95% CI**	***p* value**	**IDI**	**95% CI**	***p* value**
Baseline model	0.661	0.626–0.697	Ref	Ref	Ref	Ref	Ref	Ref	Ref
+ AIP	0.721	0.687–0.755	< 0.001	0.455	0.328 ~ 0.582	< 0.001	0.063	0.047 ~ 0.079	< 0.001
+ TC	0.678	0.644–0.713	0.039	0.159	0.029 ~ 0.289	0.016	0.013	0.005 ~ 0.020	< 0.001
+ HDL-C	0.670	0.635–0.706	0.142	0.076	-0.054 ~ 0.205	0.254	0.005	0.001 ~ 0.010	0.021
+ LDL-C	0.667	0.632–0.702	0.271	0.127	-0.003 ~ 0.256	0.056	0.004	-0.001 ~ 0.007	0.061
+ Non-HDL-C	0.685	0.651–0.720	0.013	0.252	0.123 ~ 0.382	< 0.001	0.018	0.009 ~ 0.026	< 0.001
+ CRI-I	0.697	0.662–0.731	0.001	0.281	0.152 ~ 0.410	< 0.001	0.029	0.017 ~ 0.040	< 0.001
+ CRI-II	0.676	0.641–0.711	0.046	0.194	0.065 ~ 0.324	0.003	0.011	0.004 ~ 0.018	0.003

The baseline model included age, male, BMI, current smoking, hypertension, T2DM, dyslipidemia, hs-CRP, creatinine, uric acid, FBG, HbA1c, LVEF, number of lesions, and vessels with CTO

*AUC* Area under the curve, *CI* Confidence interval, *NRI* Net reclassification improvement, *IDI* Integrated discrimination improvement, *Ref* Reference, *CRI* Castelli’s risk index, other abbreviations are as in Table 1

### Subgroup analysis

**Fig. 5:**
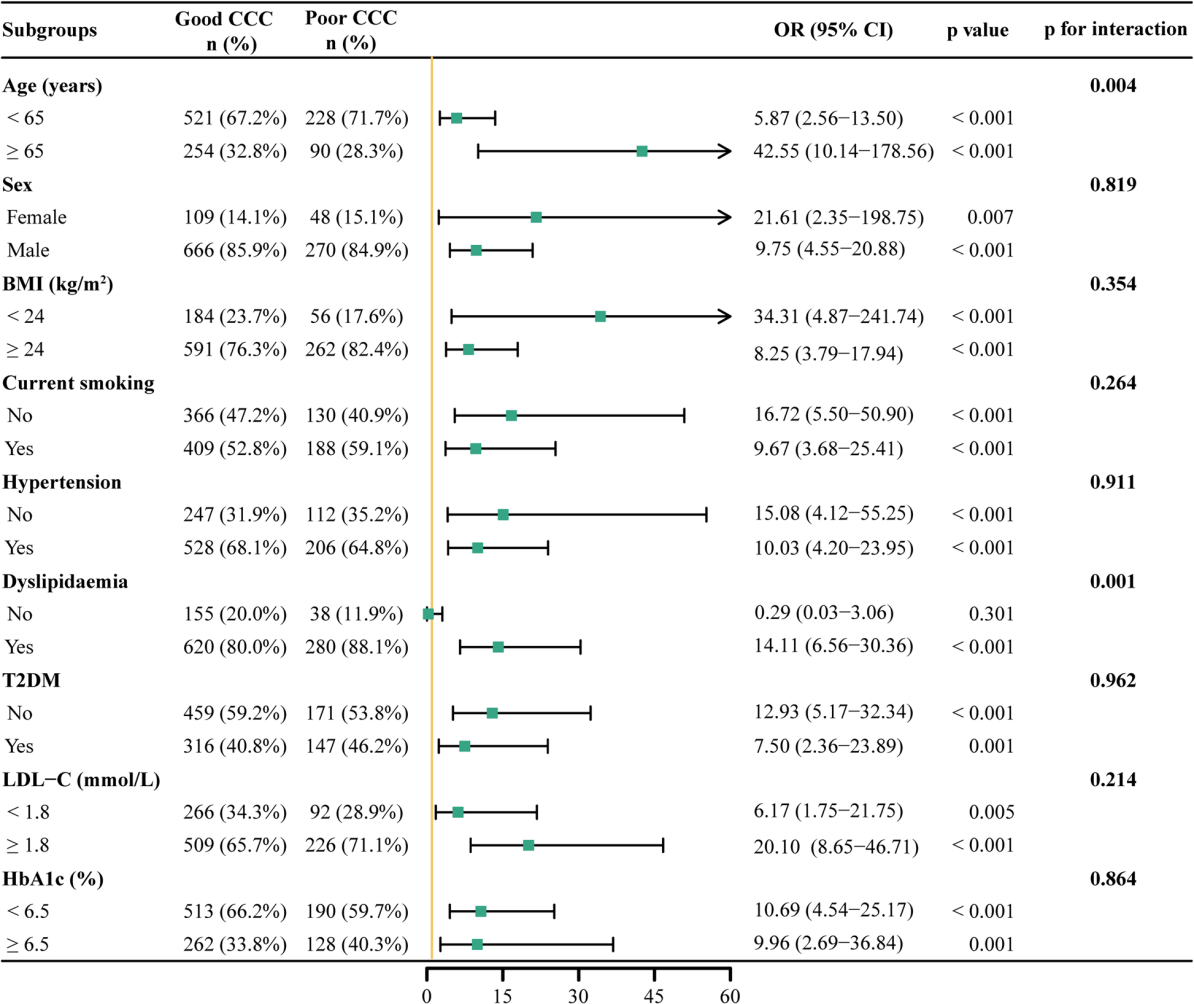
Subgroup analysis for the impact of AIP on CCC. The yellow vertical solid line represents the HR value of 1. The subgroup analysis was performed by using Model 3 (adjusted for age, male, BMI, current smoking, hypertension, T2DM, dyslipidemia, hs-CRP, creatinine, uric acid, FBG, HbA1c, TC, LDL-C, LVEF, number of lesions and vessels with CTO). AIP, atherogenic index of plasma; CCC, coronary collateral circulation; OR, odds ratio; CI, confidence interval; BMI, body mass index; T2DM, type 2 diabetes mellitus; LDL-C, low density lipoprotein cholesterol; HbA1c, glycosylated hemoglobin A1c

Subgroup analysis of the total population was performed according to age (< 65 or ≥ 65 years), sex (female or male), BMI (< 24 or ≥ 24 kg/m^2^), current smoking (yes or no), hypertension (with or without), dyslipidemia (with or without), T2DM (with or without), LDL-C (< 1.8 or ≥ 1.8 mmol/L), and HbA1c (< 6.5% or ≥ 6.5%) to further verify the utility of AIP for identifying poor CCC in different subgroups. In subgroups, excluding those without dyslipidemia, an increased AIP (per 1 unit) was consistently associated with poor CCC after adjusting for confounders with Model 3 (all *p* < 0.05) (Fig. 5). Furthermore, age and dyslipidemia were found to have interactions with AIP (all *p* for interaction < 0.05) (Fig. 5).

## Discussion

To the best of our knowledge, the present study is the first to investigate the association between AIP and CCC in CAD patients with CTO. Based on our analyses, we have the following main findings: (1) Patients with a higher AIP had a significantly higher incidence of poor CCC than those with a lower AIP. (2) AIP was independently associated with poor CCC, regardless of whether AIP was a continuous or categorical variable. (3) The inclusion of AIP in the baseline model improved the ability to identify the risk of poor CCC. (4) AIP was associated with various cardiovascular risk factors, and there was a non-linear relationship between AIP and poor CCC. In conclusion, AIP may be a potentially reliable alternative indicator for the early identification of poor CCC in CAD patients with CTO.

As anastomotic branches between epicardial coronary arteries, CCC normally only contain a small amount of blood due to their small diameter and high blood flow resistance. However, in severe epicardial coronary artery stenosis, especially in the case of CTO lesions, factors such as myocardial hypoxia and shear stress increase in the blood supply area of narrow coronary arteries, the original collateral branch can be transformed from a nonfunctional anastomotic branch to a functional anastomotic branch by expanding the diameter and thickening the wall [25]. Fulton et al. found that the caliber of CCC in non-CAD patients ranged from 10–200 μm, whereas the caliber of CCC in CAD patients could be enlarged to 100–800 μm [26]. In addition to pre-existing collateral branches, new collateral branches can form through two main mechanisms: arteriogenesis and angiogenesis. In humans, the formation of CCC is primarily driven by arteriogenesis during initial occlusion, with the dual effects of arteriogenesis and angiogenesis observed only after repeated occlusive episodes [27]. The formation of CCC is a characteristic manifestation of CTO lesions. Previous studies have shown that the incidence of functional anastomotic branches may increase from 9% in normal hearts to 95% in CTO lesions [25].

The formation of CCC can prevent or reduce ischemic myocardial necrosis, and ideally well-developed CCC can even maintain normal myocardial function in the area supplied by narrowed coronary arteries [27]. It is even considered an important indicator affecting the prognosis of patients with CAD. A meta-analysis of 12 independent studies evaluated the impact of CCC on all-cause death in patients with stable or acute CAD. They found that the mortality in the good CCC group was significantly lower than that in the poor CCC group, suggesting that good CCC is closely associated with improved prognosis [8]. Therefore, urgent research is needed to investigate the risk factors affecting the formation of CCC. Previous studies have shown that the inflammatory response and its related mediators can have adverse effects on the formation of CCC [28]. Under inflammatory conditions, the bioavailability and production of nitric oxide are impaired, leading to endothelial dysfunction and diminishing its role in promoting angiogenesis. There is also evidence suggesting that poor CCC is associated with insulin resistance, diabetes, dyslipidemia, and MetS [29].

AIP is the logarithm of the molar ratio of TG to HDL-C. It is considered a simple indicator for assessing atherosclerosis and is utilized in the evaluation of lipid-driven inflammatory states [12]. A cross-sectional study in Iran found that AIP was correlated with waist circumference, BMI, blood pressure, HDL-C, LDL-C, TG, TC, FBG, and physical activity, which is similar to our findings, suggesting that AIP could be used as a routine monitoring indicator for cardiovascular disease (CVD) risk factors [30]. In addition, numerous studies have confirmed the significant association of AIP with the occurrence, development, and prognosis of CAD. Wu et al. conducted a meta-analysis showing that a higher AIP may be independently correlated with a higher incidence of CAD [15]. According to Kim et al., AIP serves as a useful tool for identifying patients at a high risk of CV events. Their study showed that patients with higher AIP were at greater risk of future CV events and demonstrated the superiority of AIP over either TG or HDL-C alone [16]. Zheng et al. demonstrated that AIP could be used as an effective predictor of poor prognosis in non-diabetic patients with CAD after PCI [31]. A retrospective study by Qin et al., which included 2356 patients with T2DM who underwent PCI, revealed that patients with a higher AIP were significantly more likely to experience adverse CV events during 4 years of follow-up. Even after adjusting for confounding variables, the independent association between AIP and adverse CV events remained [32]. Additionally, Wang et al. further investigated the prognostic value of AIP in the population with LDL-C < 1.8 mmol/L. They exhibited that elevated AIP was significantly associated with an increased risk of major adverse cardiovascular and cerebrovascular events (MACCE) in acute coronary syndrome (ACS) patients undergoing PCI, despite having LDL-C levels well-controlled [33]. Furthermore, Zhu et al. confirmed the independent association between AIP and the risk of in-stent restenosis (ISR) in patients with ACS, especially in the LDL-C < 1.8 mmol/L subgroup [34]. The study by Süleymanoğlu M, et al. also showed that the AIP was independently associated with no-reflow in STEMI patients after primary PCI, superior to traditional lipid profiles [35]. However, there is limited research exploring the value of AIP in patients with CTO. Guelker et al. reported that AIP was related to the J-CTO score (representing the complexity of CTO), potentially helping to improve procedural planning and quality of intervention [17]. Another study suggested that AIP was significantly higher in the CTO group compared with the non-CTO group. AIP was an independent risk factor for CTO and demonstrated predictive capability for both the presence and severity of CTO [18].

Given the importance of CCC in patients with CTO [9] and the value of AIP in patients with CAD, we extended previous studies and explored the relationship between AIP and CCC in CAD patients with CTO for the first time. We found that an elevated AIP was an independent risk factor for an increased risk of poor CCC in CAD patients with CTO. Interestingly, the relationship between AIP and poor CCC in such a population is non-linear. There appears to be no positive correlation between AIP and poor CCC when AIP is below 0.18. One potential explanation is that HDL-C has a protective effect, whereas TG has an atherogenic effect, so if AIP is below the threshold, it indicates higher HDL-C and/or lower TG, which is not a sufficient risk for developing poor CCC. This could explain the absence of a significant association with poor CCC in the Q2 group, while such an association was notable in the Q3 and Q4 groups. Additionally, AIP was added to the established baseline model for analysis, revealing that its inclusion may improve the ability to identify poor CCC, which was superior to the addition of the other atherosclerotic lipid profile. However, the addition of LDL-C to the baseline model had no incremental effect, probably because of the bias caused by the use of lipid-lowering drugs. To further confirm the reliability and stability of this study, the subgroup analysis was conducted, showing that the AIP lacked good predictive value in patients without dyslipidemia. This may be attributed to the smaller sample size within this subgroup, coupled with the possibility that AIP in this subgroup was very low, failing to meet the cut-off value of AIP for identifying poor CCC.

The underlying mechanism between high AIP and poor CCC has not been fully elucidated. A possible mechanism is that AIP reflects disturbances in lipid metabolism, which impact the formation of CCC. AIP is derived from TG and HDL-C. Previous studies have shown that TG and HDL-C are independent predictors of poor CCC [10, 36]. TG is involved in the pro-inflammatory, pro-coagulant, and pro-apoptotic pathways that are crucial for atherosclerosis [37]. HDL-C can promote cholesterol efflux, inhibit vascular inflammation or oxidative stress, reduce thrombosis, and improve endothelial cell function [38]. Hypertriglyceridemia can activate cholesteryl ester transfer protein (CETP). CETP transfers TG from apolipoprotein B lipoprotein particles to HDL-C through TG exchange of cholesterol esters, resulting in a decrease in HDL-C levels and an increase in LDL-C and very low-density lipoprotein cholesterol (VLDL-C) levels in plasma [39]. High cholesterol and LDL-C levels lead to subendothelial lipid deposition, macrophage foam cell formation, plaque progression, vascular endothelial cell dysfunction, and impaired collateral vessel formation [40]. In addition, triglyceride-rich lipoprotein (TRL) and apolipoprotein B lipoprotein particles in patients with high TG levels easily form small and dense low-density lipoprotein cholesterol (sdLDL-C) through hepatic metabolism mediated by apolipoprotein C (APO-C) III [41]. SdLDL-C is considered the most atherogenic lipid index [42]. It has been shown that sdLDL-C penetrates the vascular endothelium more than LDL-C and stimulates vascular endothelial cells to secrete inflammatory mediators and adhesion molecules, resulting in vascular endothelial cell dysfunction [43]. A recent study found that AIP could serve as a substitute indicator of sdLDL-C and showed a significant negative correlation with the molecular diameter of LDL-C [44]. All of the above mechanisms between AIP and the formation of CCC need to be further confirmed.

In summary, AIP, as a comprehensive lipid index, can provide important information about the formation of CCC in patients with CTO. AIP can be used for early risk stratification of CTO patients, helping to identify high-risk patients with poor CCC, and guiding more proactive, comprehensive, and personalized treatment for these patients.

### Limitations

There are some limitations in this study. First, as a retrospective observational study, it could not prove the causal relationship between AIP and CCC in patients with CTO. Besides, there were many factors that affected AIP and CCC, and although we made extensive adjustments for possible confounders, we could not completely avoid confounding bias. Second, the patients included in this study were all from the same center, rendering unavoidable the presence of selection bias. Third, AIP was greatly affected by the levels of TG and HDL-C; however, adjustments were not made for the use, type, or dosage of lipid-lowering drugs, potentially introducing bias into the findings. Finally, the assessment of CCC did not employ the gold standard collateral flow index (CFI); instead, solely relying on the Rentrop scoring system was used. Future prospective, multicenter, large-scale studies are needed to determine the association between AIP and CCC.

## Conclusions

Elevated AIP is independently associated with an increased risk of poor CCC in CAD patients with CTO, and may improve the ability to identify poor CCC in clinical practice. Consequently, AIP can be considered a reliable surrogate for the assessment of CCC. A high AIP may serve as a reminder to clinicians that CCC in patients with CTO is likely to be poorly developed and insufficient to provide compensatory protection to the ischemic myocardium.

## Data Availability

The data used and/or analyzed during the current study are available from the corresponding author on reasonable request.

## Abbreviations

AIP: Atherogenic index of plasma
CAD: Coronary artery disease
CCC: Coronary collateral circulation
CTO: Chronic total occlusion
TG: Triglyceride
HDL-C: High-density lipoprotein cholesterol
RCS: Restricted cubic spline
ROC: Receiver operating characteristic
AUC: Area under the curve
PCI: Percutaneous coronary intervention
CABG: Coronary artery bypass grafting
MACE: Major adverse cardiovascular events
T2DM: Type 2 diabetes mellitus
MetS: Metabolic syndrome
CV: Cardiovascular
TIMI: Thrombolysis in myocardial infarction
NYHA: New York Heart Association
LVEF: Left ventricular ejection fraction
BMI: Body mass index
hs-CRP: High-sensitivity C-reactive protein
FBG: Fasting blood glucose
GA: Glycosylated albumin
HbA1c: Glycosylated hemoglobin A1c
TC: Total cholesterol
LDL-C: Low-density lipoprotein cholesterol
CRI: Castelli’s risk index
SBP: Systolic blood pressure
DBP: Diastolic blood pressure
OGTT: Oral glucose tolerance test
SD: Standard deviation
IQR: Interquartile ranges
OR: Odds ratio
CI: Confidence interval
NRI: Net reclassification improvement
IDI: Integrated discrimination improvement
LAD: Left anterior descending artery
LCX: Left circumflex artery
RCA: Right coronary artery
Ref: Reference
CVD: Cardiovascular disease
MACCE: Adverse cardiovascular and cerebrovascular events
ACS: Acute coronary syndrome
ISR: In-stent restenosis
CETP: Cholesterylester transfer protein
VLDL-C: Very low-density lipoprotein cholesterol
TRL: Triglyceride-rich lipoprotein
sdLDL-C: Small and dense low-density lipoprotein cholesterol
APO: Apolipoprotein C

## References

[1] Vaduganathan M, Mensah GA, Turco JV, Fuster V, Roth GA (2022). The global burden of cardiovascular diseases and risk: a compass for future health. J Am Coll Cardiol.

[2] Bigler MR, Seiler C. The Human Coronary Collateral Circulation, Its Extracardiac Anastomoses and Their Therapeutic Promotion. Int J Mol Sci 2019;20. 10.3390/ijms20153726 PMC6696371 31366096

[3] Gallone G, Baldetti L, Tzanis G, Gramegna M, Latib A, Colombo A (2020). Refractory angina: from pathophysiology to new therapeutic nonpharmacological technologies. JACC Cardiovasc Interv.

[4] Lee SW, Lee PH, Ahn JM, Park DW, Yun SC, Han S (2019). Randomized trial evaluating percutaneous coronary intervention for the treatment of chronic total occlusion. Circulation.

[5] Brilakis ES, Banerjee S, Karmpaliotis D, Lombardi WL, Tsai TT, Shunk KA (2015). Procedural outcomes of chronic total occlusion percutaneous coronary intervention: a report from the NCDR (National Cardiovascular Data Registry). JACC Cardiovasc Interv.

[6] Lawton JS, Tamis-Holland JE, Bangalore S, Bates ER, Beckie TM, Bischoff JM (2022). 2021 ACC/AHA/SCAI Guideline for Coronary Artery Revascularization: A Report of the American College of Cardiology/American Heart Association Joint Committee on Clinical Practice Guidelines. J Am Coll Cardiol.

[7] Seiler C (2003). The human coronary collateral circulation. Heart.

[8] Meier P, Hemingway H, Lansky AJ, Knapp G, Pitt B, Seiler C (2012). The impact of the coronary collateral circulation on mortality: a meta-analysis. Eur Heart J.

[9] Elias J, Hoebers LPC, van Dongen IM, Claessen B, Henriques JPS (2017). Impact of collateral circulation on survival in ST-segment elevation myocardial infarction patients undergoing primary percutaneous coronary intervention with a concomitant chronic total occlusion. JACC Cardiovasc Interv.

[10] Liu T, Wu Z, Liu J, Lv Y, Li W (2021). Metabolic syndrome and its components reduce coronary collateralization in chronic total occlusion: an observational study. Cardiovasc Diabetol.

[11] Shen Y, Ding FH, Dai Y, Wang XQ, Zhang RY, Lu L (2018). Reduced coronary collateralization in type 2 diabetic patients with chronic total occlusion. Cardiovasc Diabetol.

[12] Dobiásová M, Frohlich J (2001). The plasma parameter log (TG/HDL-C) as an atherogenic index: correlation with lipoprotein particle size and esterification rate in apoB-lipoprotein-depleted plasma (FER(HDL)). Clin Biochem.

[13] Won KB, Heo R, Park HB, Lee BK, Lin FY, Hadamitzky M (2021). Atherogenic index of plasma and the risk of rapid progression of coronary atherosclerosis beyond traditional risk factors. Atherosclerosis.

[14] Li YW, Kao TW, Chang PK, Chen WL, Wu LW (2021). Atherogenic index of plasma as predictors for metabolic syndrome, hypertension and diabetes mellitus in Taiwan citizens: a 9-year longitudinal study. Sci Rep.

[15] Wu J, Zhou Q, Wei Z, Wei J, Cui M (2021). atherogenic index of plasma and coronary artery disease in the adult population: a meta-analysis. Front Cardiovasc Med.

[16] Kim SH, Cho YK, Kim YJ, Jung CH, Lee WJ, Park JY (2022). Association of the atherogenic index of plasma with cardiovascular risk beyond the traditional risk factors: a nationwide population-based cohort study. Cardiovasc Diabetol.

[17] Guelker JE, Bufe A, Blockhaus C, Kroeger K, Rock T, Akin I (2020). The atherogenic index of plasma and its impact on recanalization of chronic total occlusion. Cardiol J.

[18] Liu T, Liu J, Wu Z, Lv Y, Li W (2021). Predictive value of the atherogenic index of plasma for chronic total occlusion before coronary angiography. Clin Cardiol.

[19] Galassi AR, Werner GS, Boukhris M, Azzalini L, Mashayekhi K, Carlino M (2019). Percutaneous recanalisation of chronic total occlusions: 2019 consensus document from the EuroCTO Club. EuroIntervention.

[20] Naseri K, Saadati S, Yari Z, Askari B, Mafi D, Hoseinian P, et al. Curcumin Offers No Additional Benefit to Lifestyle Intervention on Cardiometabolic Status in Patients with Non-Alcoholic Fatty Liver Disease. Nutrients 2022;14. 10.3390/nu14153224 PMC9370510 35956400

[21] Williams B, Mancia G, Spiering W, Agabiti Rosei E, Azizi M, Burnier M (2018). 2018 ESC/ESH Guidelines for the management of arterial hypertension. Eur Heart J.

[22] ElSayed NA, Aleppo G, Aroda VR, Bannuru RR, Brown FM, Bruemmer D, et al. 2. Classification and Diagnosis of Diabetes: Standards of Care in Diabetes-2023. Diabetes Care 2023;46:S19-s40. 10.2337/dc23-S002 PMC9810477 36507649

[23] Catapano AL, Graham I, De Backer G, Wiklund O, Chapman MJ, Drexel H (2016). 2016 ESC/EAS Guidelines for the Management of Dyslipidaemias. Eur Heart J.

[24] Rentrop KP, Cohen M, Blanke H, Phillips RA (1985). Changes in collateral channel filling immediately after controlled coronary artery occlusion by an angioplasty balloon in human subjects. J Am Coll Cardiol.

[25] Seiler C, Stoller M, Pitt B, Meier P (2013). The human coronary collateral circulation: development and clinical importance. Eur Heart J.

[26] Fulton WF (1963). Arterial anastomoses in the coronary circulation: II. Distribution, enumeration and measurement of coronary arterial anastomoses in health and disease. Scott Med J.

[27] Zimarino M, D'Andreamatteo M, Waksman R, Epstein SE, De Caterina R (2014). The dynamics of the coronary collateral circulation. Nat Rev Cardiol.

[28] Toprak K, Yılmaz R, Kaplangoray M, Memioğlu T, İnanır M, Akyol S, et al. Comparison of the effect of uric acid/albumin ratio on coronary colleteral circulation with other inflammation-based markers in stable coronary artery disease patients. Perfusion 2023. 10.1177/02676591231202105. 10.1177/02676591231202105 37674333

[29] Mouquet F, Cuilleret F, Susen S, Sautière K, Marboeuf P, Ennezat PV (2009). Metabolic syndrome and collateral vessel formation in patients with documented occluded coronary arteries: association with hyperglycaemia, insulin-resistance, adiponectin and plasminogen activator inhibitor-1. Eur Heart J.

[30] Niroumand S, Khajedaluee M, Khadem-Rezaiyan M, Abrishami M, Juya M, Khodaee G (2015). Atherogenic Index of Plasma (AIP): A marker of cardiovascular disease. Med J Islam Repub Iran.

[31] Zheng Y, Li C, Yang J, Seery S, Qi Y, Wang W (2022). Atherogenic index of plasma for non-diabetic, coronary artery disease patients after percutaneous coronary intervention: a prospective study of the long-term outcomes in China. Cardiovasc Diabetol.

[32] Ma X, Sun Y, Cheng Y, Shen H, Gao F, Qi J (2020). Prognostic impact of the atherogenic index of plasma in type 2 diabetes mellitus patients with acute coronary syndrome undergoing percutaneous coronary intervention. Lipids Health Dis.

[33] Wang Y, Wang S, Sun S, Li F, Zhao W, Yang H, et al. The predictive value of atherogenic index of plasma for cardiovascular outcomes in patients with acute coronary syndrome undergoing percutaneous coronary intervention with LDL-C below 1.8mmol/L. Cardiovasc Diabetol 2023;22:150. 10.1186/s12933-023-01888-3 PMC10294439 37365588

[34] Zhu Y, Chen M, Liu K, Gao A, Kong X, Liu Y (2022). Atherogenic index of plasma and the risk of in-stent restenosis in patients with acute coronary syndrome beyond the traditional risk factors. J Atheroscler Thromb.

[35] Süleymanoğlu M, Rencüzoğulları İ, Karabağ Y, Çağdaş M, Yesin M, Gümüşdağ A (2020). The relationship between atherogenic index of plasma and no-reflow in patients with acute ST-segment elevation myocardial infarction who underwent primary percutaneous coronary intervention. Int J Cardiovasc Imaging.

[36] Kadi H, Ozyurt H, Ceyhan K, Koc F, Celik A, Burucu T (2012). The relationship between high-density lipoprotein cholesterol and coronary collateral circulation in patients with coronary artery disease. J Investig Med.

[37] Tada H, Nohara A, Kawashiri MA. Serum Triglycerides and Atherosclerotic Cardiovascular Disease: Insights from Clinical and Genetic Studies. Nutrients 2018;10. 10.3390/nu10111789 PMC6266080 30453617

[38] Rye KA, Barter PJ (2014). Cardioprotective functions of HDLs. J Lipid Res.

[39] Mann CJ, Yen FT, Grant AM, Bihain BE (1991). Mechanism of plasma cholesteryl ester transfer in hypertriglyceridemia. J Clin Invest.

[40] Shen Y, Wang XQ, Dai Y, Wang YX, Zhang RY, Lu L (2022). Diabetic dyslipidemia impairs coronary collateral formation: An update. Front Cardiovasc Med.

[41] Zheng C, Khoo C, Furtado J, Sacks FM (2010). Apolipoprotein C-III and the metabolic basis for hypertriglyceridemia and the dense low-density lipoprotein phenotype. Circulation.

[42] Ikezaki H, Lim E, Cupples LA, Liu CT, Asztalos BF, Schaefer EJ (2021). Small dense low-density lipoprotein cholesterol is the most atherogenic lipoprotein parameter in the prospective Framingham Offspring Study. J Am Heart Assoc.

[43] Klisic A, Kavaric N, Vujcic S, Mihajlovic M, Zeljkovic A, Ivanisevic J (2020). Inverse association between serum endocan levels and small LDL and HDL particles in patients with type 2 diabetes mellitus. Eur Rev Med Pharmacol Sci.

[44] Viktorinova A, Malickova D, Svitekova K, Choudhury S, Krizko M (2021). Low-density lipoprotein cholesterol-to-apolipoprotein B ratio as a potential indicator of LDL particle size and plasma atherogenicity in type 2 diabetes. Diabetes Res Clin Pract.

